# Flexible Copper-Based TEM Grid for Microscopic Characterization of Aged Magnetotactic Bacteria MS-1 and Their Magnetosome Crystals in Air-Dried Droplet

**DOI:** 10.3390/molecules31020253

**Published:** 2026-01-12

**Authors:** Natalia Lorela Paul, Regis Deturche, Jeremie Beal, Catalin Ovidiu Popa, Rodica Elena Ionescu

**Affiliations:** 1 Materials Science and Engineering Department, Faculty of Materials and Environmental Engineering, Technical University of Cluj-Napoca, 400641 Cluj-Napoca, Romania; 2 Light, Nanomaterials and Nanotechnologies (L2n) Laboratory, CNRS UMR 7076, University of Technology of Troyes, 12 Rue Marie Curie, CS 42060, CEDEX, 10004 Troyes, France; 3 Eut+ Institute for Nanomaterials & Nanotechnologies EUTINN, European University of Technology, European Union

**Keywords:** magnetotactic bacteria, *Paramagnetospirillum magnetotacticum* MS-1, magnetosomes, natural magnetic nanoparticles, drop-casting method, flexible TEM grid, microscopic techniques

## Abstract

Magnetotactic bacteria (MTB) have attracted interest in recent years, mainly due to their natural ability to form intracellular magnetic nanocrystals with potential for biomedical and environmental applications. In this study, we focused on the morphological analysis of the *Paramagnetospirillum magnetotacticum* MS-1 strain, trying to keep the bacteria as close to their natural state as possible. An important element of this work is the use of untreated bacterial cells, without conductive coating or chemical fixation, using a simple and low-cost support. This choice was made intentionally to avoid changes induced by metallization and to allow direct observation of characteristics that may be relevant in applications where the interaction of the bacteria with the environment plays an important role, such as biosensors. In addition, the analysis was performed on a bacterial suspension stored for approximately 10 months at 4 °C to assess whether the morphology specific to the MS-1 strain is maintained over time. The obtained results show that the general cell morphology and magnetosome organization can be clearly and reproducibly observed even after long-term storage. Without attempting to replace studies based on conventional sample preparation methods, this work provides a complementary perspective and suggests that magnetotactic bacteria may represent a natural and effective alternative to synthetic magnetic nanoparticles, with potential applications in the biomedical and environmental fields.

## 1. Introduction

Magnetotactic bacteria represent a diverse group of prokaryotic, Gram-negative, and motile microorganisms found mainly in aquatic environments, particularly at the oxic–anoxic interface in water columns or sediments. These bacteria move using flagella and have the ability to orient themselves along the lines of the Earth’s magnetic field, a behavior known as magnetotaxis. This property gives them an important adaptive advantage, allowing them to more efficiently locate areas with optimal oxygen conditions in chemically and redox-stratified environments [1,2,3,4].

A defining feature of MTB is the presence of magnetosomes, biomineralized structures formed from magnetic nanocrystals of magnetite (Fe_3_O_4_) or greigite (Fe_3_S_4_), enclosed in a phospholipid membrane. The relatively uniform dimensions of these crystals, typically between 35 and 120 nm, give them magnetic stability and a permanent magnetic moment, making them single-domain particles. Magnetosomes are usually organized in intracellular chains, which allows bacteria to passively align with magnetic field, similar to a small magnetic needle [5,6,7,8,9]. Due to their high crystalline quality, natural biocompatibility, and magnetic stability, magnetosomes have significant advantages over synthetic magnetic nanoparticles [5,8,10].

In the context of increased interest in biological materials usable in nanotechnology, biomedicine, and environmental applications, MTBs have been proposed as natural platforms for biosensors, medical imaging, or controlled drug delivery systems [5,11,12]. However, the exploitation of these applications depends directly on a correct structural characterization of bacteria and magnetosomes. Conventional methods of sample preparation for microscopy often involve fixation, dehydration, or metallization, procedures that can alter cell morphology and surface properties, removing samples from their native state and introducing unwanted artifacts.

*Paramagnetospirillum magnetotacticum* MS-1 is a Gram-negative, microaerophilic, spiral-shaped magnetotactic bacterium that synthesizes magnetite with dimensions of approximately 45 nm [13,14,15]. The aim of this study was to characterize the morphology and structure of the MS-1 strain using a simple and accessible approach based on the analysis of untreated cells deposited on a low-cost copper-based TEM grid. Through this strategy, the paper aims to evaluate the feasibility of a rapid microscopic analysis method, designed to reflect as accurately as possible the natural state of the bacteria and to provide complementary information to studies based on classical sample preparation methods.

## 2. Results

### 2.1. SEM and STEM Analyses of Copper TEM Grid

#### 2.1.1. Untreated with MS-1 Bacterial Cells

Detailed analysis of the morphology, crystal structure, chemical composition, and organization of magnetosomes requires the use of advanced microscopic techniques that allow visualization of the bacterial surface and the distribution of magnetosomes, as well as information regarding the mapping of chemical elements or aspects related to the topography of the bacterial surface. In this regard, we analyzed the bacteria using SEM and STEM, with the aim of studying the external cellular morphology, identifying the shape of the bacteria and their size but also attempting to visualize the magnetosome chain visible on the cell surface.

In this analysis, a single TEM-grid support was used through several analysis techniques (SEM, STEM, EDX, and later AFM) to understand its characteristics and composition as a feasible support in MTB analysis.

**Figure 1:**
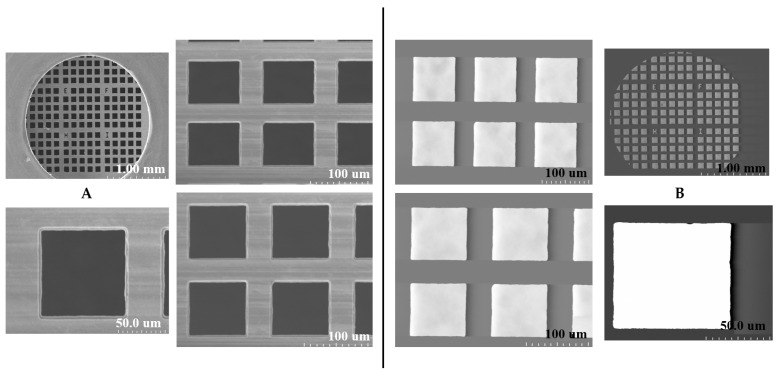
SEM (**A**) and STEM (**B**) imaging of an untreated copper-based TEM grid.

SEM and STEM investigations, as shown in Figure 1, illustrate the morphology of a copper-based TEM grid used as a sample support, before the deposition of MS-1 bacterial cells. The SEM images highlight the surface, free of contaminants or residual particles, confirming that the grid is untreated and in the absence of artifacts that could interfere with subsequent bacterial sample analysis.

#### 2.1.2. Treated with MS-1 Bacterial Cells

**Figure 2:**
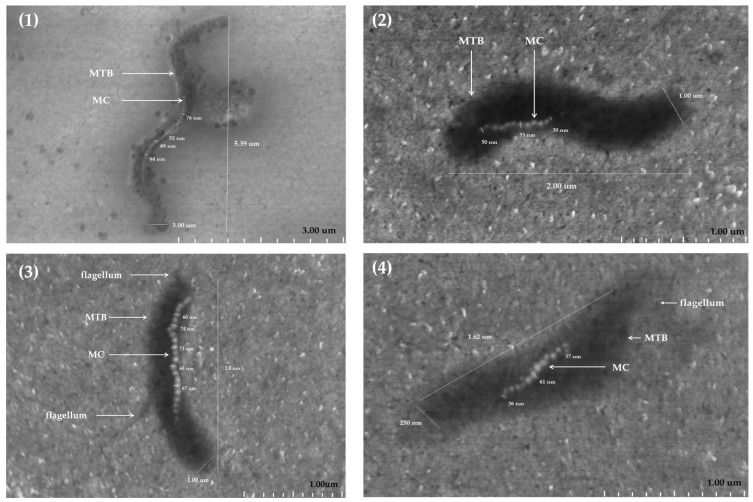
SEM morphology of four (**1**–**4**) MS-1 bacteria (sizes between 1.62–5.39 µm) and their magnetosome chains (13–34 magnetosome crystals per cell with sizes of crystals between 36 and 76 nm); MTB—magnetotactic bacteria, MS-1 strain; MC—magnetosome chain.

The analysis of MS-1 by SEM allowed the detailed characterization of the morphology of MTB and highlighted variations in their shape and size. The MS-1 bacteria have a small degree of morphological diversity; they have mainly spiral forms, but some of them are more elongated (Figure 2): morphology (1) more elongated, compared to morphology (2) with typical spiral shape. The cell surface appears relatively smooth, and at some of them it was possible to identify flagella. The dimensions of the cells analyzed by SEM vary; the length oscillates from a few hundred nanometers up to a few micrometers. As can be seen in Figure 2, the size of the bacteria varies between 1.62 and 5.39 µm, while their width varies between 250 nm and 3.00 µm. The ratio between length and width indicate the differences between elongated and more spiral-shaped bacteria, highlighting the morphological plasticity of these microorganisms.

Magnetosomes visualized by SEM also show variations in size and number in the constitution of the chains. Magnetosomal particles generally fall within the range of 30–120 nm [16,17,18], considered optimal for magnetotaxis, and the shape is predominantly cuboctahedral. The present measurements fell within the range between 30 and 76 nm for the MS-1 strain of MTB.

The distribution of magnetosome particles in chains within the same bacterial population indicates a considerable dispersion, both in chain length and in organization and distribution within the cell (13–34 magnetosome crystals per cell). This aspect can be explained by adaptation to environmental factors, such as oxygen concentration or nutritional components in the environment, which have direct implications on the organization of magnetosome chains, on magnetic coercion, and the cellular magnetic moment.

In this way, the characterization of these bacteria by SEM not only highlights the morphological and structural diversity of MTB, but also provides essential data for understanding the relationship between shape, size, and functionality within this group of microorganisms.

In STEM, images are obtained by focusing a very thin beam of electrons, which is systematically scanned along an extremely thin sample. The beam interacts with the sample, and the resulting electrons are collected by detectors, allowing an image to be formed, and can reflect both the morphological structure and the internal distribution of the material [19].

**Figure 3:**
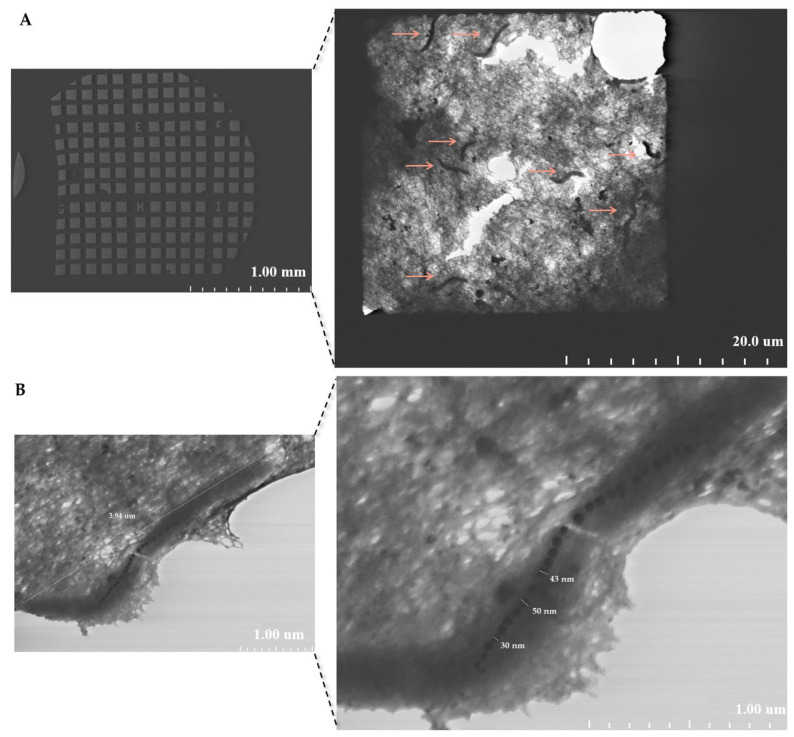
STEM image of MS-1 bacteria; (**A**)—STEM imaging of TEM grid with bacteria distributed on it, (**B**)—STEM imaging of MS-1 (length of 3.94 µm) and their magnetosome chain (28 magnetic crystals with sizes between 30 and 50 nm); bacteria indicated by orange arrows, MC—magnetosome chain.

In the present context, the copper-based TEM grid was used to assess the feasibility of this type of support in MTB, MS-1 strain analysis. The substances dissolved in the culture medium formed a compact matrix in the holes of the TEM grid, allowing STEM analysis. As can be seen in Figure 3A, several bacteria could be identified, their distribution and most importantly the dispersed way in which they are located can also be observed. The essential aspect that can be deduced is that the bacteria are found rather isolated than in clusters. The bacteria analyzed in STEM (Figure 3B) presents an elongated morphology, with a length of 3.94 µm. STEM analysis allows clear visualization of the magnetosome chain, the organized distribution inside the cell, as well as the appreciation of its length (28 magnetic crystals). The analyzed nanocrystals have dimensions between 30 and 50 nm. Thus, STEM analysis of MS-1 revealed elongated cells with a helical morphology characteristic of the genus. The images obtained allowed the clear observation of magnetosome chains arranged orderly along the longitudinal axis of the cell. These structures appeared as nanoparticles with uniform dimensions and high contrast (Figure 3).

### 2.2. EDX Analysis of a Copper TEM Grid

#### 2.2.1. Untreated with MS-1 Bacterial Cells

**Figure 4:**
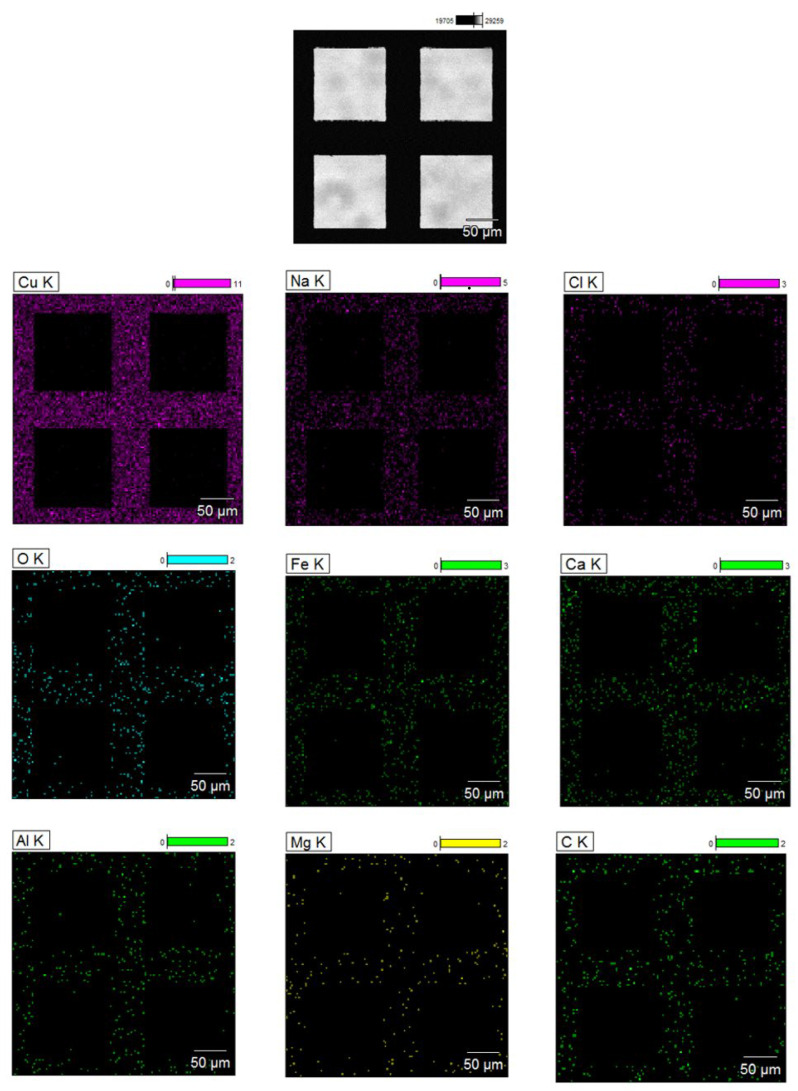
SEM image and EDX maps obtained on an untreated copper TEM grid; the square areas that appear black correspond to the gaps (mesh) in the grid, where no material is present and no EDX signal is detected. The colored areas in the EDX maps represent the solid edges of the grid, where the characteristic elements of the support are detected.

EDX analysis performed on the untreated copper grid, as shown in Figure 4, revealed a spectrum dominated by the characteristic signal of copper, confirming the basic composition of the support used. In the spectrum, moderate intensity signals for sodium and weaker signals for iron, calcium, and chlorine, as well as for carbon, oxygen, aluminium, and magnesium were also observed. The presence of carbon and oxygen can be explained by the existence of the thin carbon film of the grid and by the adsorption of compounds from the ambient air, while sodium, calcium, and chlorine suggest possible saline residues from the handling of the grid. Iron, aluminium, and magnesium can be attributed to minor contamination with metal particles or dust, occurring during the manufacturing process or handling. The low intensities of these signals indicate that they represent a minimal background, which does not compromise the subsequent interpretation of the data [20,21,22]. Establishing this elemental profile is important because it provides a reference standard for the analysis of biological samples subsequently loaded onto the grid, allowing for the clear distinction of contributions from bacterial cells and magnetosomes from possible surface contaminations.

#### 2.2.2. Treated with MS-1 Bacterial Cells

**Figure 5:**
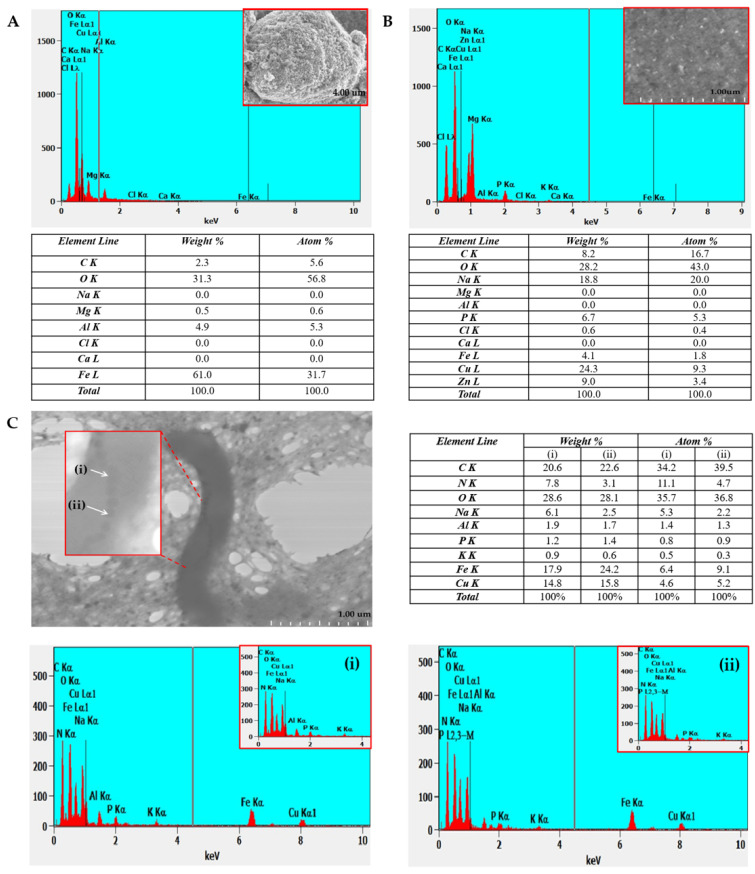
EDX spectra of (**A**)—iron oxide; (**B**)—copper-based TEM grid; (**C**)—MS-1 bacterial cell on two magnetosome crystals (**i**) and (**ii**) along with percentage of each element identified indicated in the table corresponding to each analyzed section.

EDX analysis of MS-1 allowed the determination of the elemental distribution of intracellular components, in particular magnetosomes, which contain iron-rich nanocrystals. By detecting the X-rays emitted by the sample, following electron beam excitation, EDX provided precise information on the chemical composition of intracellular structures, as shown in Figure 5. In the case of MS-1 magnetosomes, the spectrum obtained by X-ray point scan highlights the presence of iron and oxygen, confirming the controlled biomineralization of magnetite.

For a better understanding, respectively for the systematic evaluation of the composition and elemental distribution of MTB, we performed a comparative analysis between the composition of synthetic iron oxide crystals, the TEM grid, and the MS-1 magnetosomes (Figure 5A, Figure 5B, and Figure 5C, respectively).

Typically, the analysis performed on isolated crystals or on magnetite aggregates shows a ratio of approximately 70 wt% Fe and 30 wt% O, values that approach the theoretical composition of Fe_3_O_4_, where Fe represents 72.3 wt% and O 27.7 wt% [23]. Our EDX measurements on magnetite crystals confirm the expected elemental composition (Fe and O) while also showing signals from the substrate (Figure 4 and Figure 5A). On the TEM grid (copper with thin carbon film, Figure 5B), the spectra display peaks of Cu and C, as well as elements from the culture medium (Zn, Mg, P). These background contributions must be considered, as accurate identification of the sample’s elemental composition relies on careful correlation and interpretation of all detected signals.

In the points analyzed directly on the MS-1 magnetosomes (points 1 and 2—Figure 5C), the EDX spectra revealed a preponderance of iron and oxygen, confirming the nature of the nanoparticles, corresponding to magnetite. The presence of the weak phosphorus signal in these regions is associated with the magnetosome membrane, composed of phospholipids and proteins specific to the biomineralization process. In addition, the residual signals (C, Na, Cu, N, Al, K) can be attributed to the bacterial organic matrix.

EDX analysis of the non-magnetosome regions near magnetotactic bacteria, shown in Figure 5B, shows that oxygen is present in a fairly high proportion, approximately 43% atomic, while iron appears only in very small quantities, around 1.8%. In contrast, the magnetosomal regions analyzed in points 1 and 2 in Figure 5C show a completely different profile: oxygen remains high, around 36%, but iron is much more concentrated, reaching 6–9% atomic. This contrast clearly highlights that magnetosomes are the place where iron accumulates, while the surrounding areas contain only traces, supporting the idea that magnetosome formation involves a localized concentration of Fe and O, specific to magnetite. Of course, the values are not perfectly uniform (taking into account the presence of other elements), which probably reflects natural variations within the cell and/or the limitations of the EDX technique, but the trend is clear and consistent with the literature.

A study by Amor et al. [24] indicates that, although a significant proportion of cellular iron is concentrated as magnetite crystals within magnetosomes (approx. 30%), a notable fraction remains diffusely distributed in the cytoplasm or as a mobile pool of (reducible) Fe(II) and protein-bound complexes. This indicates the existence of intermediate forms or storage forms of iron involved in the controlled biomineralization process. The constant iron EDX signal in the measured points on MS-1 magnetosomes emphasizes the strict control of the biomineralization conducted by the bacteria, and the homogeneous distribution of iron. All these findings confirm the data in the literature regarding the partial storage of iron in the composition of magnetosomes and its diffuse presence in the cytoplasm [24].

Overall, the combined SEM/STEM-EDX analysis confirms that the magnetosomes produced by MS-1 are predominantly composed of iron oxides, their ordered organization in chains being a consequence of the rigorous biological control exercised during biomineralization. The elemental distribution on MS-1 reflects the precise compartmentalization of the biochemical processes involved in the formation and stabilization of these nanocrystals. The EDX method thus offers a powerful combination of structural and chemical analysis and contributes to the understanding of how MS-1 magnetotactic bacteria internally organize their magnetosomes to achieve optimal magnetotactic behavior.

### 2.3. AFM Analysis of a Copper TEM Grid

#### 2.3.1. Untreated with MS-1 Bacterial Cells

**Figure 6:**
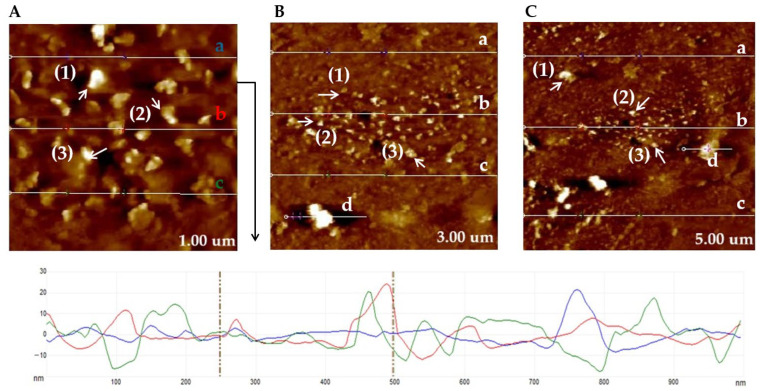
AFM measurements of untreated copper-based TEM grid in the intersection regions between meshes, images of the surface measured of the TEM grid at 1.00 µm (**A**), 3.00 µm (**B**), and 5.00 µm (**C**) (the particle size corresponding to the grid is marked with Arabic numerals, and the sections corresponding to the RMS are marked by letters). Note: AFM measurements indicate that, for the 1 µm grid, the RMS of the analyzed sections (a–c) varies between 2.069, 4.868, and 8.362 nm (the colored letters (a–c) correspond to the colored lines in the image below), while the copper grain particle sizes measured in regions 1–3 are 57.6, 70.1, and 48.6 nm, respectively. In the case of the 3 µm grid, the RMS values (a–d) range from 2.54, 7.69, 2.72, and 7.65 nm across the investigated sections, whereas the particle sizes (1–3) vary between 83.7, 134.8, and 146.5 nm. For the 5 µm grid, a higher surface heterogeneity is observed, with RMS values (a–d) between 4.92, 6.90, 13.27, and 7.98 nm, accompanied by significantly larger particle sizes (1–3) of 314.7, 143.8, and 152.8 nm. RMS—Root Mean Square.

AFM images in height sensor mode obtained on the untreated copper TEM grid, as shown in Figure 6, at different scan sizes (1 µm, 3 µm, and 5 µm), highlight the characteristic surface morphology of the support. The topography shows a rough surface (average RMS value of 5.09 nm, 4.31 nm, 8.36 nm at 1 µm, 3 µm, and 5 µm, respectively). Thus, the asperities are relatively homogeneously distributed, associated with the natural microtexture of the carbon film and the granulation of the copper layer (copper grain diameter of 58.7 nm, 121.6 nm, 203.7 nm at 1 µm, 3 µm, and 5 µm, respectively). Analysis of the profile sections (from left side to right side) shows height variations of the order of a few nanometers up to a few hundred nanometers, which confirms the absence of macroscopic contaminants and residual deposits (Figure 6).

The AFM measurements reveal the topography without prominent foreign particles, confirming that the grid is untreated and suitable for use as a reference support in subsequent real sample experiments. These results establish a control profile of surface roughness and morphology, which can be used for comparison with samples loaded with MS-1 magnetotactic bacteria and their magnetosomes.

#### 2.3.2. Treated with MS-1 Bacterial Cells

**Figure 7:**
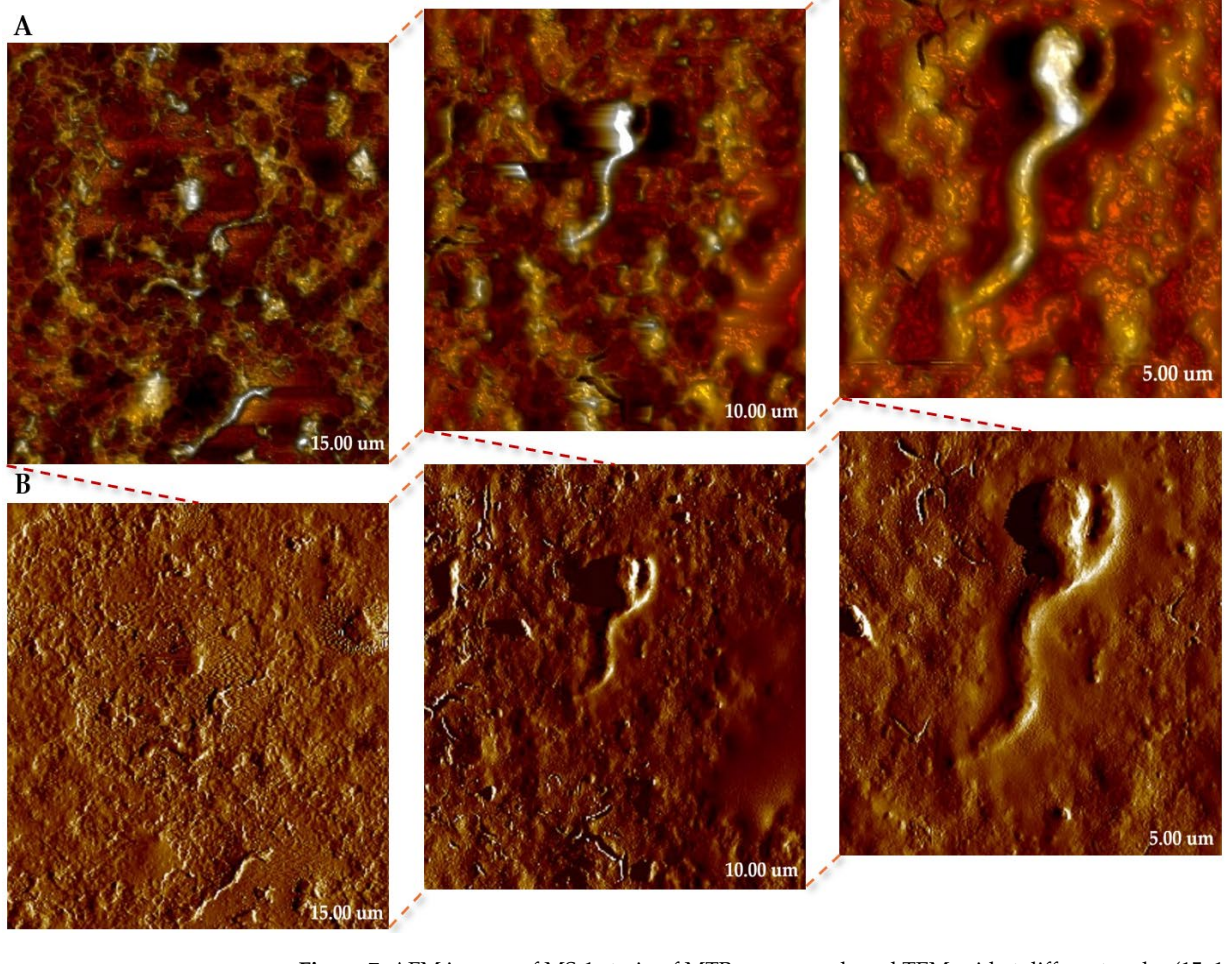
AFM images of MS-1 strain of MTB on copper-based TEM grid at different scales (15, 10, and 5 µm, respectively); (**A**)—height sensor images; (**B**)—peak force error images.

To obtain a detailed insight into the morphology and topography of the surface of bacteria, AFM analyses were performed on MS-1. The images obtained provided a faithful representation of the cellular structure and highlight the specific organization of these magnetotactic microorganisms. In Figure 7, we present the topography (in height sensor mode—Figure 7A) and peak force error channel images (Figure 7B), both acquired simultaneously during the scan. The height sensor images provide real height values, but the visual representation may lose clarity in areas where the geometry of the bacteria is highly distorted. On the other hand, the Peak Force Error signal highlights rapid changes in relief, producing a light–shadow contrast that highlights fine surface details, difficult to distinguish from the topography alone. It is important to emphasize that peak force error has an exclusively illustrative role, not being suitable for morphometric quantifications, for which only the images obtained in height sensor are used [25,26,27,28].

**Figure 8:**
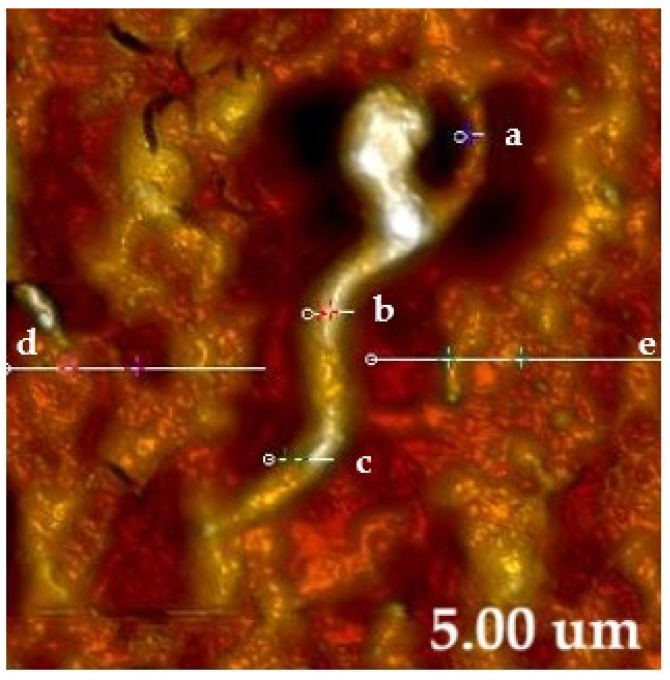
AFM analysis of copper-based TEM grid treated with MS-1 (bacteria size 3.78 µm). Note: For the MS-1 cell section (5 µm), the RMS values measured at three points (at the ends and central portion of the bacteria: (a–c) are 11.20, 21.15, and 13.64 nm, respectively. In the surrounding background matrix, the RMS measured in regions (d,e) is 13.53 and 11.97 nm. RMS refers to root mean square roughness.

The AFM image obtained on MS-1 (Figure 8) highlights the specific topography of an elongated cell, with spiral morphology, characteristic of the genus Magnetospirillum. The cell presents a continuous, homogeneous surface, without obvious discontinuities, which indicates good structural preservation during the analysis of the sample. The topographic analysis allows the estimation of a length of the bacterium of approximately 3.78 µm, a value consistent with the data obtained by analyzing the bacterium using SEM and STEM techniques.

The topographic contrast reveals fine variations in the surface relief, attributed to the internal chain of magnetosomes aligned along the longitudinal axis, which determine small changes in the locally measured height. Thus, the average roughness values obtained for the bacterial surface indicate a slightly wavy texture, associated with the internal distribution of magnetosomes and ultrastructural variations of the cell membrane (values of 11.20 nm and 13.64 nm at the bacterial ends, and 21.15 nm in the central portion, respectively). In comparison, the RMS values of the background matrix, representing the solidified medium in which the bacteria are suspended, are smaller and relatively close (13.53 nm and 11.97 nm), which confirms the clear delimitation between the bacterial surface and the supporting substrate. This difference between the roughness of the bacteria and that of the matrix highlights the fidelity of the AFM measurements and allows the correlation of the observed morphological details with the internal structure specific to magnetotactic bacteria.

## 3. Discussion

In this study, a long-term stored bacterial suspension was used instead of a freshly isolated one to evaluate how well MS-1 bacteria retain their structural and magnetic properties over time. This approach provided us with essential information about their long-term functional stability and potential relevance in practical applications, where resistance to different environmental conditions is critical.

Sample preparation typically involves fixation, dehydration, and coating with a conductive layer (gold, or carbon), procedures that can alter the native structure of the cells and modify the surface appearance, generating morphological artifacts that are difficult to distinguish from real features [29,30,31,32]. Also, because MTB are microaerophilic or strict anaerobic bacteria, their exposure to oxygen during sample preparation can affect cell viability and the integrity of intracellular compounds, including magnetosomes that are sensitive to environmental chemical conditions [29,31,33,34,35]. The authors proposed the standalone analysis of the MS-1 strain of MTB, without prior coating with a conductive layer. The reasoning and motivation in this regard stemmed from the observation of the limited number of publications performing SEM characterization of MTB strains [36,37,38], especially on the MS-1 strain [15,39,40].

Analysis of the MS-1 strain using advanced microscopy (SEM, STEM, EDX, and AFM) provided comprehensive insight into the morphology, structure, and chemical composition of the bacteria. Each method provided complementary information, but most importantly, it confirmed the feasibility of the low-cost TEM metal substrate used in these experiments. SEM provided information on the external morphology, shape, and distribution of magnetosomes [29,41,42] without using a conductive coating, which allowed the bacteria to be observed as close to their natural state as possible. STEM complemented these data by visualizing the internal structure and alignment of the magnetosome chains [43,44,45]. EDX confirmed the chemical composition of the magnetosomes and highlighted the difference between the signals coming from the substrate and those from the bacteria [46,47,48,49,50,51,52]. AFM was useful for analyzing the topography of the substrate and the deposited bacteria, demonstrating the efficient attachment of the cells and the real differences from the substrate background [53,54].

The combined results show that copper-based TEM supports can be used for MS-1 characterization, with certain limitations. SEM, STEM, and EDX allow faithful reproduction of the investigated features, but protocols must be adapted to experimental conditions. AFM can be used, but the presence of substrate roughness can hinder topographic analysis.

Overall, the study highlighted both the advantages and limitations of using this type of low-cost substrate in the microscopic analysis of marine magnetotactic bacteria, emphasizing the novelty of the work: the identification and characterization of bacteria in a state as close as possible to their natural state, using an accessible and reproducible method.

## 4. Materials and Methods

### 4.1. Bacterial Culture

*Paramagnetospirillum magnetotacticum* MS-1 (DSMZ:DSM 3856) grown in standard culture medium provided by the supplier contains 100 μM of Fe(III) quinate solution, seven-vitamin solution, and mineral solution [55,56]. We used an aged bacterial suspension (10 months at 4° C), not a fresh or newly isolated one, in order to investigate and highlight their stability over time.

### 4.2. Support and Sample Preparation

**Figure 9:**
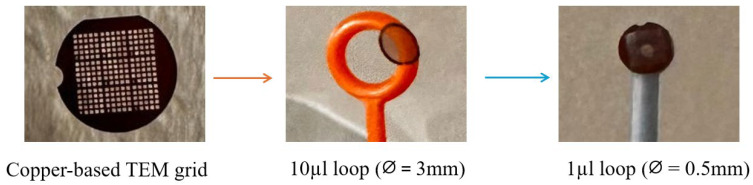
Copper-based TEM grid manipulation.

A drop of MS-1 bacterial suspension (equivalent to 5 µL) was added to the assay support, a copper-based TEM grid, 200 mesh grids (pitch 127 µm, hole width 90 µm, bar width 37 µm) (Ted Pella. Redding, CA, USA). In order to prevent possible contamination when handling, the TEM grid was sampled using a sterile loop. Initially, a 10 µL loop (3 mm diameter) was used. However, due to the larger diameter of the loop, it was adjusted using a 1 µL loop (diameter of 0.5 mm); see Figure 9.

### 4.3. Microscopic Characterization

MS-1 bacteria were directly adsorbed onto a copper-based TEM grid for SEM, STEM, and EDX analyses. Imaging was performed using a Hitachi FEG SU8030 scanning electron microscope (Hitachi High-Technologies Corporation, Tokyo, Japan) under vacuum conditions ranging from 5 to 25 kV, with a working distance of 10–15 mm. AFM analysis was performed on bacteria adsorbed on copper-grid TEM. Images were obtained with a Bruker, Dimension Icon atomic force microscope (Bruker Corporation, Billerica, MA, USA). The measurements were carried out in air, in ScanAsyst mode, using a silicon tip on a nitride lever and a cantilever with k of 0.4 N/m, f0 of 70 kHz.

## 5. Conclusions

The integrated application of advanced microscopic techniques (SEM, STEM, EDX, and AFM) on the strain *Paragmnetospirillum magnetotacticum* MS-1 allowed obtaining a complex and complementary picture of the morphology, composition, and organization of bacteria and their magnetosomes. SEM and STEM provided detailed information on the structure of the cells and the arrangement of the magnetosomal chains, EDX confirmed the presence of key elements (especially Fe and O) associated with magnetite crystals, and AFM highlighted the dimension and contour of the cell at the nanometric scale, completing the structural data. The results support the idea of a controlled biomineralization process, characteristic of MTB, and also reveal the existence of intracellular fractions of non-crystallized iron, with relevance for understanding bacterial metabolism.

The use of low-cost copper-based TEM grid support has proven relatively effective for cross-analysis by all four techniques, representing an accessible and reproducible solution for morphological, structural, and elemental studies, with the condition that a rigorous analysis of the signals coming from the substrate is performed.

Beyond the methodological value, these advanced analyses contribute to the consolidation of the role of MTB as sensitive bioindicators and functional nanostructured materials, given their sensitivity to variations in the redox environment and their ability to reflect changes in iron and oxygen concentrations. Thus, the use of several advanced microscopy techniques for characterizing MS-1 not only expands the understanding of the biological mechanisms of biomineralization but also provides valuable information for biomedical and biosensors applications, as well as for ecological surveillance and assessment of the health of ecosystems.

## Data Availability

Data are contained within the article.
